# A dynamic causal model for evoked and induced responses

**DOI:** 10.1016/j.neuroimage.2011.07.066

**Published:** 2012-01-02

**Authors:** Chun-Chuan Chen, Stefan J. Kiebel, James M. Kilner, Nick S. Ward, Klaas E. Stephan, Wei- Jen Wang, Karl J. Friston

**Affiliations:** a Wellcome Trust Centre for Neuroimaging, Institute of Neurology, University College London, UK; b Graduate Institute of Biomedical Engineering, National Central University, Taiwan; c Max Planck Institute for Human Cognitive and Brain Sciences, Leipzig, Germany; d Sobell Department of Motor Neuroscience and Movement Disorders, Institute of Neurology, University College London, UK; e Laboratory for Social and Neural Systems Research, Institute for Empirical Research in Economics, University of Zurich, Switzerland; f Department of Computer Science and Information Engineering, National Central University, Taiwan

## Abstract

Neuronal responses exhibit two stimulus or task-related components: evoked and induced. The functional role of induced responses has been ascribed to ‘top-down’ modulation through backward connections and lateral interactions; as opposed to the bottom-up driving processes that may predominate in evoked components. The implication is that evoked and induced components may reflect different neuronal processes. The conventional way of separating evoked and induced responses assumes that they can be decomposed linearly; in that induced responses are the average of the power minus the power of the average (the evoked component). However, this decomposition may not hold if both components are generated by nonlinear processes. In this work, we propose a Dynamic Causal Model that models evoked and induced responses at the same time. This allows us to explain both components in terms of shared mechanisms (coupling) and changes in coupling that are necessary to explain any induced components. To establish the face validity of our approach, we used Bayesian Model Selection to show that the scheme can disambiguate between models of synthetic data that did and did not contain induced components. We then repeated the analysis using MEG data during a hand grip task to ask whether induced responses in motor control circuits are mediated by ‘top-down’ or backward connections. Our result provides empirical evidence that induced responses are more likely to reflect backward message passing in the brain, while evoked and induced components share certain characteristics and mechanisms.

## Highlights

► In this study, we propose a Dynamic Causal Model for evoked and induced responses. ► We use this model to study the relationship between evoked and induced responses. ► We find that induced responses are mediated by backward connections in motor circuits. ► This model is important for disambiguating the top-down and bottom-up process.

## Introduction

Neuronal activity exhibits a broad range of event-related electromagnetic oscillations (Crone et al., 1998a, 1998b; Grosse et al., 2002; Kilner et al., 2003). Event-related cortical oscillatory activity can be divided into evoked and induced components (Galambos, 1992; Tallon-Baudry and Bertrand, 1999). Evoked and induced responses are elicited by endogenous or internal changes (e.g. a thought) or exogenously (e.g. a stimulus) or both. Operationally, the difference between evoked and induced responses is their phase-relationship to a timed event, such as a presented stimulus. Specifically, evoked components are phase-locked to the stimulus, whereas induced responses show trial-to-trial variations in latency. A growing number of studies have demonstrated that induced responses increase with cognitive demand; such as attention, expectation, learning and perception, especially in gamma-band range (30–70 Hz) (Chen et al., 2009; Gilbert and Sigman, 2007; Kaiser and Lutzenberger, 2003; Lee et al., 2003; Tallon-Baudry et al., 1997). The functional role of induced responses has therefore often been interpreted as reflecting ‘top-down’ modulation through backward or lateral connections; as opposed to the bottom-up driving processes that may be more manifested in evoked components mediated by forward projections (Tallon-Baudry and Bertrand, 1999). The implication is that the evoked and induced responses may reflect different neuronal processes and mechanisms. However, a recent study using simulated data has reported that the evoked and induced responses may “share” common generative mechanisms, up to a certain level (David et al., 2006). For example, if there are amplitude variations in stimulus-locked inputs (“dynamic causes”), the evoked power will be recapitulated in the induced power as the variance of the amplitude increases; although the evoked responses remain the same (see David et al., 2006 for details). One possible explanation for these amplitude variations (gain effects) is the effect of attention (McAdams and Maunsell, 1999; Treue and Martinez-Trujillo, 1999). Moreover, the detection of induced responses relies upon careful comparisons between carefully matched experimental and control conditions (Kaiser and Lutzenberger, 2003) and the results may be confounded by uncontrolled factors contributing to experimental conditions. For instance, attention is rarely an all-or-none event; rather, attention is engaged in a graded fashion (Gilbert and Sigman, 2007; Joseph, et al., 1997). Therefore, a generic model, which accounts for the generating mechanisms shared by both induced and evoked responses, may be necessary when inferring the underlying neuronal processing. Indeed, the conventional way of separating evoked and induced responses assumes that they can be decomposed in a linear fashion (see Tallon-Baudry and Bertrand, 1999 for an example). This linear assumption is questionable given the evidence that various empirically observed neuronal (population) responses, such as sustained oscillatory dynamics, are difficult to explain without invoking context-dependent or nonlinear mechanisms (Deco et al., 2008)

In this paper, we propose a generic dynamic causal model (DCM) (Chen et al., 2008; Friston et al., 2003; Kiebel et al., 2009) that can explain both evoked and induced responses jointly in a single experimental dataset. This generic model uses bilinear state equations to model spectral density dynamics (Chen et al., 2009), in terms of a single neuronal architecture; however, it tries to explain both evoked and induced responses (obtained through different pre-processing of the same data), simultaneously, as different trial types or conditions. In this way, condition-specific changes in coupling strength reflect the mechanisms associated with induced responses that cannot be explained by the evoked ones. In other words, this approach allows the direct study of the relationship between the evoked and induced neural activity with respect to the underlying generative mechanisms. Critically, our model explicitly accounts for nonlinear neuronal mechanisms that are expressed in terms of cross-frequency coupling (Chen et al., 2008)

To establish the face validity of this model, two synthetic data sets were created to test the identifiability of various generating mechanisms using Bayesian Model Selection (BMS). Having established the face validity of the scheme we applied it to an empirical MEG dataset to ask whether induced responses in the motor control circuits are mediated by ‘top-down’ or backward connections. We illustrate this application using data from a hand-grip paradigm, which have been used previously to address a fundamental issue about nonlinearities in neuronal networks in the motor system (Chen et al., 2010). On the basis of our previous findings, we tested three models (forward, backward and forward-backward) which differ in the connections that are modulated by the induced condition, relative to evoked. This paper is organised as follows: in the next section, the generic model used in this work will be reprised briefly. This is followed by a description of the simulation studies used to validate the scheme and the empirical (MEG motor study) data used to illustrate its application. The final section presents the results of the simulated and empirical analyses.

## Materials and methods

### A generic model of evoked and induced responses

This model is exactly the same as the DCM described in Friston et al. (2003) and Chen et al. (2008), but is recapitulated here to highlight how we model the difference between evoked and induced responses. The underlying state equations of this DCM describing the motion of spectral density measures *g*(*ω*, *t*) = [*g*_1_(*ω*, *t*), …, *g*_*n*_(*ω*, *t*)]^*T*^ in *n* sources have a bilinear form:

1 $$\tau \dot{g}=(A+vB)g+Cu\text{.}$$

The matrices *A* and *C* contain coupling parameters that control changes in spectral activity induced by other sources and exogenous (e.g., stimulus) inputs, *u*(*t*). The matrices *B* are introduced to encode the coupling changes induced by the condition effects, *v* ∈ {0, 1}. In our particular application, the condition effects represent whether the data features reflect evoked (*v* = 0) or induced (*v* = 1) responses, for any particular trial type. Evoked and induced ‘conditions’ are created during pre-processing by performing the time-frequency analysis after (evoked) and before (induced) trial averaging. As in conventional DCM for induced responses, the coupling matrices decompose into:

2 $$\begin{array}{cc} A_{\mathrm{ij}}=\left[\begin{array}{ccc} a_{\mathrm{ij}}^{11} & \cdots & a_{\mathrm{ij}}^{1K}\\ \vdots & \ddots & \vdots \\ a_{\mathrm{ij}}^{K1} & \ldots & a_{\mathrm{ij}}^{\mathrm{KK}} \end{array}\right] & B_{\mathrm{ij}}=\left[\begin{array}{ccc} b_{\mathrm{ij}}^{11} & \cdots & b_{\mathrm{ij}}^{1K}\\ \vdots & \ddots & \vdots \\ b_{\mathrm{ij}}^{K1} & \ldots & b_{\mathrm{ij}}^{\mathrm{KK}} \end{array}\right]\;C_{i}=\left[\begin{array}{c} c_{i}^{1}\\ \vdots \\ c_{i}^{K} \end{array}\right] \end{array}\text{.}$$

Under this model, the scalar *a*_*ij*_^*kl*^ encodes how changes in the *k*-th frequency in the *i*-th source depend on the *l*-th frequency in the *j*-th source. The leading diagonal elements are *a*_*ii*_^*kk*^ = − 1; this means that each frequency has an intrinsic tendency to decay or dissipate. Similarly, *c*_*i*_^*k*^ controls the frequency-specific influence of exogenous inputs on the *k*-th frequency in the *i*-th source. Together, this parameterization enables coupling due to linear (within-frequency) and nonlinear (between-frequency) mechanisms within and between sources (for details, see Chen et al., 2008). In this generic model the *A* coupling matrices model the ‘shared’ influences mediating both the evoked and induced activities, while the *B* matrices model influences that are specific to induced response components. Note that we ignore in Eq. (2), for readability, the trial type index for matrices *B*.

### Simulated data

**Fig. 1:**
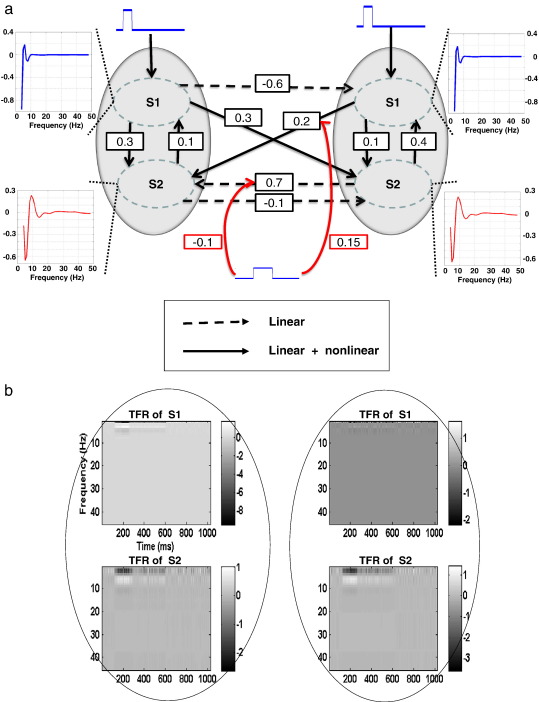
(a) Simulation architecture and parameters. The two grey circles represent the two areas in this model, while S1 and S2 denote two frequency modes within each area. A frequency mode corresponds to a pattern of frequency-specific deviations from the baseline spectral profile (these patterns are shown as a function of frequency in the inserts). Time-dependent modulations of these frequency modes correspond to evoked and induced responses. The solid lines represent nonlinear connections because they connect different frequency modes, while the dashed lines couple the same frequency modes and therefore model linear coupling. The red lines indicate the connections that can change in a condition-specific fashion (here, whether we are trying to explain induced or purely evoked spectral responses). (b) The time-frequency data generated by this model. These are linear mixtures of the two time-varying frequency modes above. Left column: Area 1; right column: Area 2.

The goal of the simulations was to test whether our scheme can correctly recognise the underlying mechanisms generating induced and evoked response components. In particular, we wanted to see if we could explain the two components in terms of a (phenomenologically) plausible model of spectral dynamics. To this end, two synthetic datasets were generated using the model in Eq. (1) and the distributed source architecture described below. These datasets differed in terms of whether their generation mechanisms specific for induced responses or not. The first (ER + IR) set allowed the induced-condition to switch on selected B parameters; while the second (ER) used B = 0. Fig. 1 shows the model architecture and the parameters used to generate the ER + IR dataset. The example in this figure comprises two areas, where the spectral dynamics of each area are modelled as a time-varying mixture of two frequency profiles or modes (S1 and S2), whose profiles are shown in the insets. These profiles were based on empirical results from a previous study (Chen et al., 2010). Fluctuations in time-frequency responses about the baseline are modelled in terms of these modes, whose dynamics depend on the coupling within (linear) and between (nonlinear) modes, within (intrinsic) and between (extrinsic) areas (see figure legend for details). In this model, we allowed for both linear and nonlinear connections; and changes in these connections to explain induced components (above and beyond evoked components). Three test models were then used to invert both synthetic datasets. The first (ER + IR) model, allowed changes in the B parameters, while the second and the third models precluded these bilinear (induced) effects. The second (ER1) model, has the ‘correct’ priors on the *A* matrices, which were identical to the connections used to generate the data (see Table 1 and Fig. 3a for these connections). The third (ER2) model allows for non-zero values in all *A* connections. The ER2 model was used to test if preventing optimum changes in the *B* parameters leads to the discovery of some ‘false’ connections. Using BMS, we hoped to show that the scheme could disambiguate among the three competing models properly.

### Empirical data

#### Experimental protocol and pre-processing

Nine healthy, right-handed (mean age 26, range 20–32 years of age) subjects participated in this study. Written consent was obtained from all subjects, in accordance with the Declaration of Helsinki. Some of these data have been reported in Chen et al. (2010). We briefly summarise the experimental protocol here. Subjects were instructed to perform a visually cued ballistic isometric grip, using their dominant hand with an inter-trial interval of 7 ± 2 s. Prior to scanning, subjects were asked to grip a manipulandum to generate a maximum voluntary contraction (MVC) and then were trained to approximate a target force (45% of MVC) with visual feedback. During scanning, no visual feedback of force was provided. This design tries to engage modulation/supervision mechanisms in the motor system as well as to minimise activity in occipital and parietal sources. Force output was recorded using a MEG-compatible gripper and was used to identify movement onset (i.e. the reaction time, from the onset of the visual cue until the onset of the ballistic grip), the grip duration and force level. MEG signals were measured continuously at 240 Hz during task performance using a whole-head CTF Omega 275 MEG system. At the beginning and end of each measurement, the positions of three anatomical landmarks (bilateral pre-auricular points and nasion) were recorded to exclude excessive head movement (thresholded at 1.5 cm and the measured maximal translation across subjects < 1.3 cm; 2.68–12.68 mm).

**Fig. 2:**
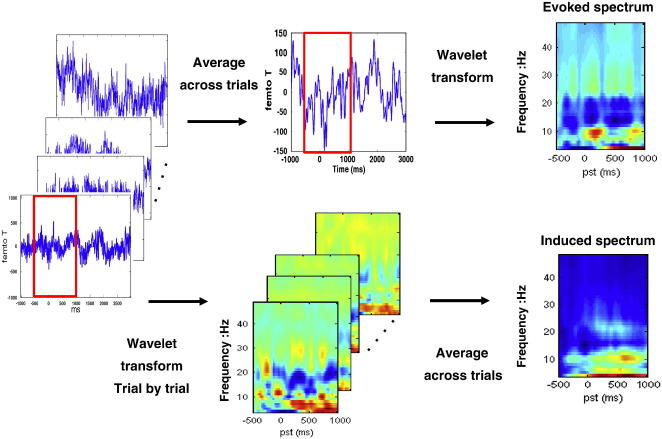
The flowchart of data preparation for evoked (upper) and induced (lower) responses. The red rectangles represent the time window of interest from − 500 to 1000 ms. Note that the spectral densities have been normalised individually.

The MEG data were pre-processed offline using SPM8 (Wellcome Trust Centre for Neuroimaging, http://www.fil.ion.ucl.ac.uk/spm/). The data were epoched from − 500 to + 1000 ms, where time zero indicates movement onset. Poorly performed (reaction times of more than one second) and artefact contaminated (MEG amplitude > 500 fT) trials were excluded from further analysis; resulting in 88–98 artefact-free epochs (88 98 90 98 94 96 90 93 95) across subjects, with 642.66 ± 54.92 ms mean reaction time and 639.45 ± 54.48 ms grip duration. The mean force level was 37.73 ± 20.27% of subject-specific MVC, suggesting that the subjects followed the instructions and performed the task well. Artefact-free epochs were averaged across trials to compute evoked responses. Then, both the individual artefact-free epochs and their average (the evoked response) were projected from channel space to source space using the generalised inverse of the lead-field matrix for our chosen sources (see Model specification below). The spectral density from 4 to 48 Hz at each source was estimated over peri-stimulus time using a time-frequency Morlet wavelet transform (wavelet number: 7). The frequency ranges cover the theta (4–8 Hz), alpha (8–15 Hz), beta (15–30 Hz), and gamma (> 30 Hz) bands. The absolute values of the resulting time-frequency responses were averaged over artefact-free epochs to produce the induced response. The corresponding transform of the trial averaged data constituted the spectral density of the evoked response. Baseline power was removed by subtracting the frequency-specific power at the first time-bin to furnish the evoked and induced conditions that were subsequently modelled. Fig. 2 illustrates this data preparation. For computational expediency, we reduced the dimensionality of spectra into four principal frequency components derived from a Singular Value Decomposition of the spectra that are subject-specific. This procedure accounts for the large inter-individual variability of frequencies seen in the motor system (Aoki et al., 2001; Kilner et al., 2003; Kristeva et al., 2007; Omlor et al., 2007) and preserved over 93% of the spectral variance in all subjects (range 93%–97%). The resulting spectral dynamics of evoked and induced components are the observations that the model is trying to explain.

### DCM specification (sources and coupling)

**Fig. 3:**
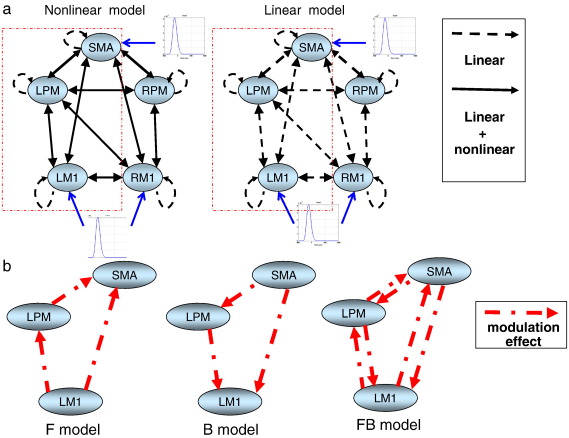
Model specifications of Forward (F), Backward (B), and Forward-Backward (FB) models based on the previous MEG study (Chen et al., 2010). (a) The basic network configuration has a left hemispheric dominance and extrinsic nonlinear connections (left) and an ‘all-linear’ model (right) is included to test whether nonlinear mechanism is crucial to explain the dataset, in particular, the induced responses. The blue arrows specify the areas which receive the exogenous input perturbation. The exogenous input was modelled using a gamma function with two normally distributed parameters estimated from the data. (b) The modulation effects are allowed in the left hemisphere (red rectangle) in only forward (F model), or backward (B model) or both forward and backward connections (FB model).

The source locations for modelling the empirical data were taken from the group results of an fMRI study using an identical task; where five subjects performed 25 ballistic isometric hand grips to 45% of MVC (for details, see Ward et al., 2008). The locations were taken as the peak coordinates in the Montreal Neurological Institute (MNI) space within each significant cluster (voxels significant at p < 0.05, corrected for multiple comparisons across the whole brain), including bilateral primary motor cortex, (M1; [− 41 − 26 56] and [49 − 27 56]), bilateral premotor cortices (PM; [− 30 − 8 64] and [46 0 58]) and left supplementary motor area (SMA; [− 2 − 2 62]). In addition, right M1 was included because of significant task-related deactivation during hand grip (Ward et al., 2008). Using these five sources and, from our previous Bayesian Model Selection result on the best connectivity architecture (see Chen et al., 2010 for details and Fig. 3a; left), we specified the three different models shown in Fig. 3b. These models test where the induced effects arise, in terms of interactions (connections) among sources. We wanted to test whether the induced responses are mainly mediated by backward (B) or forward (F) or both (FB) connections in the motor network (Fig. 3b). We focused on the induced (bilinear) effects in the left hemisphere, since this is a right hand movement task (Fig. 3a; left; red rectangle). In these models, the SMA is assumed to be higher in the motor hierarchy than PM and MI, as suggested by studies in which the Bereitschaftspotential (BP; or readiness potential/field) has been measured; these studies suggest that the SMA is involved in the planning and initiation of movement (Deecke, 1987, 1990; Keller and Heckhausen, 1990; Praamstra et al., 1995; Shibasaki and Hallett, 2006). In addition, we add an ‘all-linear’ model (Fig. 3a; right) based on the winning model of the three comparing models specified above to test whether nonlinear mechanism is crucial to explain the dataset, in particular, the induced responses.

### Inference on models

The testing DCMs were inverted (fitted) for each subject. To identify the best models at the group level, we compared the log evidences or marginal likelihoods between models (Penny et al., 2004), after pooling over subjects under fixed effect assumptions. This assumes that all subjects use the same model. In addition, we employed random effects BMS (Stephan et al., 2009) to accommodate inter-individual variability in the structure of models or functional architectures that gave rise to subject-specific brain activity during our task.

### Inference on the parameters of the winning model

Subject-specific estimates of the modulation matrices Bij (see Eq. (2)) from all subjects, under the best model identified by BMS, were smoothed (to account for inter-subject variability in frequency-to-frequency coupling) using a Gaussian kernel with a Full-width half-maximum of 8 Hz. These matrices summarise the frequency-to-frequency coupling associated with each connection. Statistical tests were applied to each element of these coupling parameter matrices to establish the significance of coupling over subjects (in relation to intersubject variability) using classical inference. This can be regarded as a standard summary statistic approach to random effects inference, using the posterior coupling estimates as subject-specific summary statistics. The corresponding SPMs of the T-statistic (thresholded at p < 0.005 uncorrected) were computed for ‘excitatory’ (positive) and ‘inhibitory’ (negative) effects respectively.

## Results

### Simulation results

Table 1 summarises our simulation results. It can be seen that using BMS, the ER + IR and the ER1 models were identified as the best models when using the ER + IR and ER data, respectively. Note that BMS is based on the log-evidence (*F*), which considers both accuracy and complexity of competing models (Stephan et al., 2009). Our model selection results are thus not confounded by the higher complexity of ER + IR relative to ER1.

Evaluating the quality of our parameter estimates in terms of mean squared error (MSE), showed that the best models (in terms of model evidence) also had the minimal MSE (9.08% and 33.51%, respectively) among the models tested (see Table 1). In addition, it is evident that the ‘left-out effect’ in the *B* matrices does not necessarily lead to inference on ‘false’ connections, as the ER2 model does not explain the ER + IR data better. Similarly, for the ER data, both ER + IR and ER2 models have a lower model evidence even though they entail more parameters than the ER1 model. This result provides another example of the well-known fact that a more complex model is not necessarily a better model (Chen et al., 2009, 2010).

Altogether our simulation results thus instil confidence that our DCM correctly disambiguates evoked and induced components of event-related responses in terms of their generative mechanisms. In the next step, we applied the model to our empirical MEG data.

### Experimental results

#### Inference on model space

**Fig. 4:**
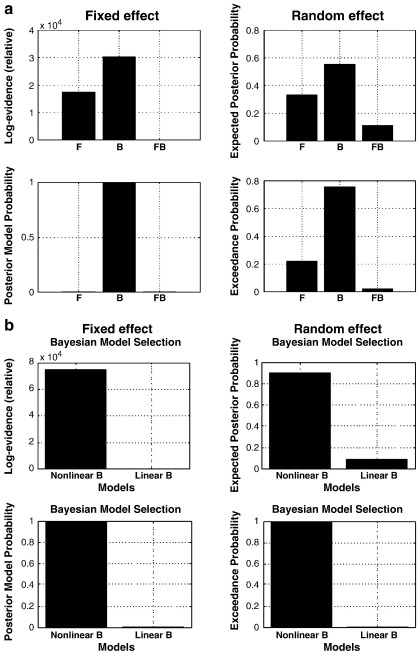
(a) Bayesian Model Selection results of nonlinear models at the group level under fixed effects (left) and random effects (right) assumptions suggest that the B (Backward) model is the winning model among the models tested. (b) Bayesian Model Selection between linear and nonlinear B models shows that nonlinear B model is superior to the linear B model and supported the idea that nonlinear connectivity is essential to the generating of induced responses.

Firstly, three DCMs were inverted for each subject as described above. Bayesian Model Selection supported the B model (with modulations of backward connections): the summed log-evidences over subjects (under fixed effects assumptions) were − 255490, − 242540 and − 272900 for F, B and FB model, respectively. This means that Bayesian Model Selection identified the B model (relative log-evidence = 30358; posterior model probability > 0.99) as the best model, given the data (Fig. 4a, left), followed by the F and FB models. An additional random effects analysis gave equivalent results, choosing the B model as the best model with an exceedance probability (i.e., probability of the selected model being more likely than all other models) of 0.7598, followed by the F model (exceedance probability 0.2211) and the FB model (exceedance probability 0.091) (Fig. 4a, right). In other words, when accounting for between-subject variability in model structure (for example, subject-dependent cognitive strategies), the model with induced components mediated by backward connections (the B model) was superior to the other two models, whereas the FB model was clearly the worst. As in the above simulations, note that the most complex model (here, the FB model) did not turn out to be the best model.

Having established the best model, B model, we then further test if an “all-linear” backward model is sufficient to explain the data. In other words, we wanted to examine whether the nonlinear connections are important to the induced responses. It can be seen in Fig. 4b that the previous winning B model (with nonlinear connections; termed nonlinear B) remained superior to the linear B model and supported the idea that nonlinear connectivity is essential to the generating of induced responses.

#### Inference on coupling parameters

**Fig. 5:**
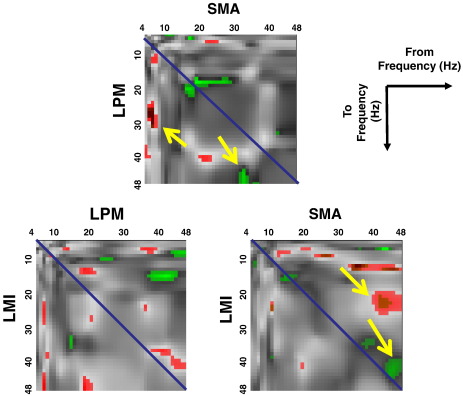
The corresponding SPMs (T-statistic map; thresholded at p < 0.005 uncorrected) of the connection-specific modulation matrices for significant ‘excitatory’ (positive; red blobs) and ‘inhibitory’ (negative; green blobs) effects respectively. The yellow arrows indicate several instances of consistent nonlinear interactions in the backward connections across all subjects.

Fig. 5 shows the corresponding SPMs (T-statistic map; thresholded at p < 0.005 uncorrected) of the bilinear (induced) matrices for significant ‘excitatory’ (positive; red blobs) and ‘inhibitory’ (negative; green blobs) effects respectively. As seen in Fig. 5, we found several instances of consistent nonlinear interactions in the backward connections across all subjects. For example, low frequency oscillations (< 10 Hz) in SMA facilitate the beta rhythm (20–30 Hz) in LPM; and the gamma rhythm (30–40 Hz) in the SMA has an inhibitory effect on the gamma (> 40 Hz) rhythm in LPM (Fig. 5; upper panel; yellow arrows). A further example can be seen in the modulatory interactions between SMA and LMI, in which the gamma rhythm (> 40 Hz) from SMA enhances the beta rhythm (20–30 Hz) and suppresses the gamma rhythm in the LMI (Fig. 5; lower panel; right; yellow arrows).

## Discussion

### Model specification in DCM

A question central in hypothesis-driven modelling like DCM is whether it is possible to identify a ‘true’ or ‘correct’ model architecture. This is a difficult question (see Stephan et al., 2010 for a detailed discussion). First, a model is, by definition, a simplification of real world complexity; for this reason there is no such thing as a ‘true’ model (cf. Box and Draper, 1987). One can, however, establish a ground truth artificially by specifying a model and generating synthetic data sets (with added noise), asking whether the known model architecture can be inferred from the noisy simulated data. This is the approach taken here (and in previous work on DCMs of different data types, e.g. Chen et al., 2008; David, in press; Moran et al., 2009; Reyt et al., 2010; Stephan et al., 2008), with reassuring results. A second problem is that even if a ‘true’ model existed, it may not be included in the set of alternative hypotheses one is comparing. As discussed in Stephan et al. (2010), since there are an infinite number of model alternatives, all one can do is “motivate model space carefully”, using prior knowledge of the problem and the neuronal system in question. It is generally helpful to specify and test the important dimensions in a model space systematically; for instance, in a factorial fashion (Chen et al., 2009; Chen et al., 2010; Daunizezau et al., in press; Stephan et al., 2007). In our simulations, we have shown that BMS can select the optimal model from the tested alternatives. For example, when we compare the ER + IR, ER1 and ER2 models, given the (noisy) ER data, BMS correctly chooses the ER1 model (Table 1; right column). In other words, adding more connections and making the model more complex (as in the ER2 model) do not make this model superior. This is because in DCM, the causality (i.e. temporal precedence) entailed by the connections is embedded in the (differential) state equations and, under optimisation of the log-evidence, these connections must be able to explain the data both in a parsimonious and accurate fashion. Therefore, by increasing model complexity too much (i.e., adding more connections than needed), the evidence of the model diminishes.

Clearly, although our simulations were based on biologically plausible parameter values from previous empirical studies (Chen et al., 2010), they were rather limited in scope. They are presented as a sufficiency proof of face validity, rather than an exhaustive exploration of parameter space. We imagine that people could repeat these tests of face validity for any architecture (DCM) under consideration, using the procedures that we have described.

### Possible mechanism of evoked and induced responses

A wealth of neuroanatomical evidence suggests that backward connections are more modulatory in relation to the driving effects of forward connections (Angelucci et al., 2002a, b; Lamme et al., 1998; Murphy and Sillito, 1987; Salin and Bullier, 1995; Sandell and Schiller, 1982). Furthermore, the underlying generative mechanisms of backward connections are likely to be nonlinear (Chen et al., 2009, 2010; Salin and Bullier, 1995; Sherman and Guillery, 1998). Combined with cognitive findings (Galambos, 1992; Tallon-Baudry and Bertrand, 1999), it has been suggested that induced responses are an expression of these top-down modulatory effects mediated by backward connections. In this study, we report that the backward connections from higher to lower areas mediate induced responses in the motor system that cannot be explained by forward connections. As noted by one of our reviewers, because DCM tries to explain responses in dynamical terms, it supports systems-level interpretations: For example, the re-entrant backward connections of the DCM allow longer responses (that persist through recurrent message passing). This may therefore provide an explanation for induced responses that typically last longer than evoked response (see Fig. 2). These additional backward influences are expressed in our DCM as induced components of event-related activities. This is in line with the notion of top-down modulation and partially dissociates the nature of induced from evoked responses. Importantly, as this task is pre-programmed in the brain during the training phase, this backward modulatory coupling is in consistent with active inference and predictive coding (Friston, 2010; Rao and Ballard, 1999) that the planned (predicted) movement representation in the higher level (i.e. SMA and/or PM) influences the motor predictions in the lower M1 level.

The most prevalent hypothesis about evoked responses is that they reflect bottom-up driving processes mediated by forward connections and employ mainly linear mechanisms. This had been seen at the mesoscopic scale where the propagation of signals through the cell layers of the cortex is a linear phenomenon (Yamawaki et al., 2008). Furthermore, the functional properties of forward connections are predominantly, but not exclusively, linear; see Friston et al. (2003) and Sherman and Guillery (1998) for a summary of the neurophysiological evidence. However, evidence is emerging for nonlinear coupling in forward connections in several networks at the system level (Chen et al., 2009, 2010). In these studies, we found evidence that forward connections also employ nonlinear mechanisms (in terms of cross frequency coupling).

Recent studies of event-related potentials (i.e. evoked responses) suggest that backward connections are essential in explaining the late ERP components in mismatch negativity studies (Garrido et al., 2007, 2009). Taken together, both empirical and simulation data (David et al., 2006) suggest that evoked and induced responses may rest on common mechanisms that generate both components to facilitate the functional integration among brain areas. Evoked and induced components may share certain characteristics, but induced responses may depend more heavily on top-down or backward connections. Further investigation of the frequency-specific coupling in forward and backward connections may help to differentiate the neuronal mechanisms that give rise to evoked and induced responses.

## Conclusion

In this study, we present a generic scheme for Dynamic Causal Modelling of evoked and induced responses. This scheme accounts for the shared mechanisms generating evoked and induced responses and allows the direct study of their relationship in terms of cortico-cortical coupling. Using simulations and BMS, we were able to show that the true generative model can be correctly identified. The empirical findings reported in this paper suggest that induced responses are more likely to be mediated by backward or top-down connections in motor circuits, while accepting that evoked and induced components are generated by some common mechanisms.

## Figures and Tables

**Table 1:** Simulation parameters and results (signal-to-noise ratio = 13.8 dB).

Values used to generate data	ER + IR data $A=\left[\begin{array}{cccc} \lambda & 0.3 & -0.6 & 0.3\\ 0.1 & \lambda & 0 & 0.1\\ 0 & 0.2 & \lambda & -0.1\\ 0 & 0.7 & 0.4 & \lambda \end{array}\right]$ $B=\left[\begin{array}{cccc} \lambda & 0 & 0 & 0\\ 0 & \lambda & 0 & 0\\ 0 & 0.15 & \lambda & 0\\ 0 & -0.1 & 0 & \lambda \end{array}\right]$	ER data $A=\left[\begin{array}{cccc} \lambda & 0.3 & -0.6 & 0.3\\ 0.1 & \lambda & 0 & 0.1\\ 0 & 0.2 & \lambda & -0.1\\ 0 & 0.7 & 0.4 & \lambda \end{array}\right]$
Estimates using the ER + IR model	$A=\left[\begin{array}{cccc} \lambda & 0.325 & -0.661 & 0.239\\ 0.097 & \lambda & 0 & 0.059\\ 0 & 0.105 & \lambda & -0.021\\ 0 & 0.638 & 0.3975 & \lambda \end{array}\right]$ $B=\left[\begin{array}{cccc} 0 & 0 & 0 & 0\\ 0 & 0 & 0 & 0\\ 0 & 0.138 & 0 & 0\\ 0 & -0.029 & 0 & 0 \end{array}\right]$ F = − 3613.2; MSE = 9.08%	$A=\left[\begin{array}{cccc} \lambda & 0.257 & 0.411 & 0.240\\ 0.101 & \lambda & 0 & 0.032\\ 0 & 0.036 & \lambda & 0.002\\ 0 & 0.552 & 0.142 & \lambda \end{array}\right]$ $B=\left[\begin{array}{cccc} 0 & 0 & 0 & 0\\ 0 & 0 & 0 & 0\\ 0 & 0.012^{} & 0 & 0\\ 0 & -0.010^{} & 0 & 0 \end{array}\right]$ F = − 3862.5; MSE = 71.13%
Estimates using the ER1 model	$A=\left[\begin{array}{cccc} \lambda & 0.161 & 0.410 & 0.100\\ 0.103 & \lambda & 0 & 0.051\\ 0 & 0.074 & \lambda & -0.027\\ 0 & 0.685 & 0.165 & \lambda \end{array}\right]$ F = − 3645.3; MSE = 51.83%	$A=\left[\begin{array}{cccc} \lambda & 0.338 & -0.135 & 0.523\\ 0.080 & \lambda & 0 & 0.009\\ 0 & 0.016 & \lambda & -0.023\\ 0 & 0.579 & 0.403 & \lambda \end{array}\right]$ F = − 3855.1; MSE = 33.51%
Estimates using the ER2 model	$A=\left[\begin{array}{cccc} \lambda & 0.176 & 0.478 & 0.123\\ 0.098 & \lambda & 0.205^{} & 0.041\\ 0.014^{} & 0.031 & \lambda & 0.026\\ 0.076^{} & 0.123 & 0.564 & \lambda \end{array}\right]$ F = − 3653.6; MSE = 85.37%	$A=\left[\begin{array}{cccc} \lambda & 0.328 & -0.024 & 0.278\\ 0.139 & \lambda & -0.048^{} & -0.019\\ 0.035^{} & -0.065 & \lambda & -0.009\\ 0.030^{} & 0.203 & 0.059 & \lambda \end{array}\right]$ F = − 3902.4; MSE = 77.64%
